# The Mutability of Yeast Prions

**DOI:** 10.3390/v14112337

**Published:** 2022-10-25

**Authors:** Chih-Yen King

**Affiliations:** Institute of Molecular Biology, Academia Sinica, Taipei 115, Taiwan; cking@imb.sinica.edu.tw

**Keywords:** prion strain, mutability, [*PSI*], *SUP35*, Hsp104, amyloid, chaperone, yeast

## Abstract

Prions replicate by a self-templating mechanism. Infidelity in the process can lead to the emergence of new infectious structures, referred to as variants or strains. The question of whether prions are prone to mis-templating is not completely answered. Our previous experiments with 23 variants of the yeast [*PSI*^+^] prion do not support broad mutability. However, it became clear recently that the heat shock protein Hsp104 can restrict [*PSI*^+^] strain variation. This raises the possibility that many transmutable variants of the prion may have been mistaken as faithful-propagating simply because the mutant structure was too sturdy or too frail to take root in the wild-type cell. Here, I alter the strength of Hsp104 in yeast, overexpressing wild-type Hsp104 or expressing the hypo-active Hsp104^T160M^ mutant, and check if the new environments enable the variants to mutate. Two variants hitherto thought of as faithful-propagating are discovered to generate different structures, which are stabilized with the hypo-active chaperone. In contrast, most transmutable variants discovered in cells overexpressing Hsp104 have been correctly identified as such previously in wild-type cells without the overexpression. The majority of transmutable variants only mis-template the structure of VH, VK, or VL, which are the most frequently observed variants and do not spontaneously mutate. There are four additional variants that never give rise to different structures in all cell conditions tested. Therefore, quite a few [*PSI*^+^] variants are faithful-propagating, and even the transmutable ones do not freely evolve but can only change to limited structural types.

## 1. Introduction

Prion aggregates are amyloid fibers where polypeptides adopt a common planar folding pattern and stack up periodically [1,2]. Polypeptide chains in adjacent stacks are in parallel and are connected by main-chain hydrogen bonds to form a ß-sheet-rich structure; identical residues in each polypeptide are in register. The fiber grows by recruiting the cellular prion protein to the two ends and templating its structural conversion to the same planar fold. Fibers are fragmented by host disaggregation machinery to generate more seeds for the templated growth, thereby facilitating cellular propagation. Like viruses, prions also exhibit strain variation. The same prion protein can assume different planar folding patterns to stack up. Each fiber type can replicate faithfully and interact with the host uniquely to cause distinctive phenotypes. 

Structures can change in interspecies infection of a prion variant, where the seed interacts with a homologous protein with amino acid substitutions that create steric hindrances, causing templating errors [3,4,5,6]. Mis-templating could also happen when the seed and the substrate have the same amino acid sequence and chemical modifications (homotypic seeding). Here, the error is likely due to some weak kinetic barriers along the substrate folding pathway that are unable to efficiently block partially converted intermediates from going astray under thermal and environmental noise. It is not clear how frequently mis-templating occurs in homotypic seeding. The mammalian prion PrP^SC^ is thought to exist as a cloud of structures, implying the process of templated structural conversion is error-prone [7,8,9]. In contrast, TDP-43 filaments derived from different brain regions of an amyotrophic lateral sclerosis (ALS) patient and from different individuals suffering from the disease all have the same structure, as revealed by high-resolution cryo-EM studies, suggesting more faithful templating [10]. The key question is whether the mis-templating is a general property of amyloids, intrinsic to the “parallel in-register” architecture, or whether it only occurs with selected fiber types. The question derives practical relevance from the fact that mis-templating offers a way for prions to evolve, thus enabling novel properties to emerge in infected humans and animals. 

Yeast prions provide favorable systems for rigorous analysis of structural transmutation in amyloids. Convenient yeast genetic tools allow for confident identification of new prion variants, ensuring that a new phenotype emerging from a clone is not due to confounding changes in the cell background. Strain variation has been reported for several yeast prions, including [*PIN*^+^], [URE3], and [*PSI*^+^], which are composed of the Rnq1, Ure2, and Sup35 proteins, respectively [11,12]. This study focuses on the latter. The Sup35 protein is a subunit of the yeast translation termination factor. It contains an N-terminal prion-forming domain, rich in glutamine and asparagine residues, a middle domain that is less well understood, and a C-terminal domain, performing the essential function of translation termination. When forming prion, Sup35 molecules are sequestered in [*PSI*^+^] aggregates. This reduces the effective cellular concentration of the protein, thus compromising translation termination and causing the suppression of nonsense mutations (nonsense suppression). Different [*PSI*^+^] structures may sequester Sup35 to different extents, thereby showing different nonsense suppression efficiencies.

Many laboratories have isolated [*PSI*^+^] variants [5,6,13,14,15]. Our earliest effort led to the identification of three variants, namely VH, VK, and VL [14]. It turned out that VH was likely the original [*PSI*^+^] (“ur-psi”) discovered by Cox in 1964 [16], and VK was identical to the first known variation, named [*ETA*^+^], reported a decade before the landmark paper by Wickner, identifying the [*PSI*^+^] element as a prion [11,17,18]. The Sc4 and Sc37 variants, reported in 2004, were also shown to be identical to “ur-psi”/VH and [*ETA*^+^]/VK, respectively [15,18]. We subsequently improved experimental methods for inducing and distinguishing [*PSI*^+^] variants and were able to discover 20 more of them in the wild-type yeast background [18]. The refined variant typing method is used in this work and is described in detail in Materials and Methods. 

Experiments with the 23 [*PSI*^+^] variants seemed to indicate that the fidelity of homotypic seeding was template-dependent [18]. Bateman and Wickner suggested that a single [*PSI*^+^] isolate is a mixture (cloud) of inter-converting amyloid conformers. The relative proportion of the conformers drifts randomly from cell to cell, causing mitotic descendants of a yeast clone to exhibit varied abilities in transmitting [*PSI*^+^] to tester strains in cytoduction experiments [19]. We have transmitted four stable variants, namely VH, VK, VL, and W8, to the same set of testers and found no evidence of structural drift in mitotic cells [20]. There are also three very transmutable variants, named UnS, Kbar, and B6, which spontaneously turn into VH and VK, VK and VL, and VK, respectively. Other variants either appear faithful-propagating or infrequently mis-template to generate VH, VK, or VL in mitosis [18]. Despite the clear-cut observation, recent progress in understanding how Hsp104 influences prion selection, described below, prompts me to re-investigate the mutability of [*PSI*^+^] variants. 

The Hsp104 protein is the yeast disaggregase that severs [*PSI*^+^] fibers [21]. It is composed of six identical subunits, forming a helical-like structure with a central channel, where substrates enter. The disaggregase hydrolyzes ATP molecules and changes conformation, thereby doing work on a bound substrate [22,23]. Deficiency in Hsp104 activity impairs fiber fragmentation, causing some mitotic cells to inherit no prion seeds at all. Hsp104 overexpression also cures [*PSI*^+^], likely due to excessive trimming from fiber ends, which leads to complete dissolution [24] (see ref. [25] for a different model). Therefore, a right balance of Hsp104 activities is critical for [*PSI*^+^] maintenance [26,27]. We recently obtained three new [*PSI*^+^] variants (in addition to the 23 variants mentioned above, which were isolated in wild-type cells), named V2, V3, and V4, in yeast expressing a hypo-active Hsp104 mutant (Hsp104^T160M^) [26]. The variants regularly mis-template VH, VK, and VL structures, which are nevertheless too sturdy to be efficiently fragmented by the mutant Hsp104. As a result, the mis-templated structures can hardly propagate and the infidelity is obscured. Conversely, the stronger Hsp104 in wild-type cells that support VH, VK, and VL dissolves V2, V3, and V4 (V2-V4). (VH, VK, and VL do not mis-template V2-V4.) It is possible that there are intrinsically transmutable [*PSI*^+^] variants not yet recognized as such. They only appear faithful-propagating because mis-templated structures cannot gain a foothold in the cell, being either too sturdy for fragmentation by Hsp104 or too weak to survive its trimming. 

Here, I re-examine the mutability of the 23 [*PSI*^+^] variants. The variants are transmitted to mutant cells expressing Hsp104^T160M^ as well as to wild-type cells overexpressing Hsp104. I then determine if a new variant can emerge and be selectively retained in the altered environments. 

## 2. Materials and Methods

### 2.1. General Yeast Methods

Experiments were performed with the 74-D694 yeast background (*ade1-14*(*UGA*) *ura3-52 leu2-3,112 trp1-289 his3-*∆*200*; ref. [21]). Genetic manipulations were performed according to Sherman [28]. Hygromycin B, Geneticin (G418), cycloheximide, and 5-fluoroorotic acid (5-FOA) were used at concentrations of 200, 400, 10, and 750 mg/L, respectively. Centromere-based plasmids, YCp33(*URA3*) and YCp111(*LEU2*), and 2µ-based plasmids, YEp195(*URA3*) and YEp181(*LEU2*), were used as vectors to carry yeast genes [29].

### 2.2. Hsp104 Overexpression

YEp195-KanMX-Cup1-Hsp104 and YEp181-KanMX-Hsp104 were used to overexpress Hsp104. The former contained a G418 selection marker, *kanMX* [30], followed by a 500-bp copper-inducible *Cup1* promoter (nucleotide -3 to -502 of *CUP1-1*), a *Bam*HI restriction site, and the *HSP104* coding sequence plus 278-bp 3′-UTR; the latter contained the selection marker and the *HSP104* gene, including 483-bp 5′-UTR and the 3′-UTR. CuSO_4_ at 50 µM final concentration was used to induce gene expression via the *Cup1* promoter.

### 2.3. Cytoduction

The cytoplasmic donor for the S17R* and D1 variants was the wild-type cell, also carrying the [*PIN*^+^] prion. The donor for other variants was [*pin*^-^], had *SUP35* deleted from the chromosome, and the gene was carried instead by YCp111-KanMX-I-SupF, containing the G418 selection marker, followed by a 1.2-kb *SUP35* promoter, a *Bam*HI restriction site, and the coding sequence plus 1.1-kb 3′-UTR. Recipient cells expressing Hsp104^T160M^ were described [26]. To facilitate reverse cytoduction, they were further made uracil-prototrophic by homologous recombination with a wild-type *URA3* gene fragment. Cytoduction recipients ([*pin*^−^]) carried the *kar1∆15* allele for karyogamy interruption and the cycloheximide-resistant *cyh2* allele for counter-selection, and were cured of mitochondrial DNA. Procedures for genetic modification are described [26]. 

Prion-free recipient cells were pre-transformed with YCp111-KanMX (for T160M cells) or YEp181-KanMX-Hsp104 (for overexpressing Hsp104) and then mixed with [*PSI*^+^] donors on YPD plates for 24 h at 30 °C. (The KanMX marker facilitated subsequent variant-typing procedures.) Cells were streaked on YPD plates containing cycloheximide to select against donor cells and diploids. After 2 days at 30 °C, cells were replica-plated onto YPG to select for cytoductants, which received healthy mitochondria from the donor. Colonies were randomly picked and arranged on YPD + G418 for variant typing. Selected cytoductants were tested for a lack of YCp111-KanMX-I-SupF, which could be transferred from the donor on rare occasions. This was done by *Bam*HI digestion of a PCR fragment spanning the *SUP35* promoter and part of the coding sequence. The DNA amplified from the plasmid was cut, but that from the chromosome was not. 

For reverse cytoduction, the above-mentioned donor cell deleted of chromosomal *SUP35* was cured of [*PSI*^+^] and mitochondrial DNA and then used to receive cytoplasm. After mixing with the new donor, it was counter-selected on synthetic complete media supplemented with 5′-FOA, then replica plated onto YPG to isolate cytoductants. 

### 2.4. Serial Propagation

Two cytoductants for each variant (or two clones of the only D1 cytoductant overexpressing Hsp104) were streaked on YPD + G418 to isolate single colonies. For each cytoductant, five lighter-colored (non-red) single colonies (or colony sectors) were selected and variant-typed. One of the five colonies was streaked again to select five sub-colonies for variant typing (5 × (2 lineages) = 10 colonies total). The procedure was repeated several times. Colonies of all shades of pink were selected. Before the experiment, the cytoductants were re-confirmed to have the correct *HSP104* genotype. 

### 2.5. Induction of Variants 5 and 6

T160M cells ([*PIN*^+^]) were doubly transformed with YEp195-Cup1-Sup35(1-253)(S17R)-GFP and YCp111-KanMX. The former carried the coding sequence of the Sup35(S17R) fragment fused in frame with GFP followed by a 240-bp *SUP35* 3′-UTR, and the latter provided a G418 marker to facilitate subsequent manipulations in variant typing. Transformants were grown in 3 mL of synthetic complete media, lacking uracil and leucine (SC-Ura, Leu), but containing 50 μM of CuSO_4_ for [*PSI*^+^] induction. After 48 h at 30 °C, cultures were streaked on YPD + G418 plates. Light-colored colonies were picked and transferred to fresh plates, replica-plated to SC-Leu plates containing 5-FOA to lose the inducing plasmid, and then replica-plated again to YPD + G418. 

### 2.6. Variant Typing

[*PSI*^+^] is most easily observed in yeast containing a nonsense mutation in the *ADE1* or *ADE2* gene, such as the *ade1*-14 or *ade2-1* allele. Each gene encodes an enzyme essential for adenine synthesis. The nonsense mutations block the synthesis, causing an upstream intermediate to accumulate and turn into a red pigment, which colors the yeast colony [31]. Cells (*ade1*-14 or *ade2-1*) without [*PSI*^+^] form red colonies. [*PSI*^+^] recruits Sup35 into the aggregation, thus lowering its effective cellular concentration and elevating stop codon readthrough. Which, in turn, reduces the accumulation of the red pigment, making colonies white or pink. 

Mutations in the N-terminal prion-forming domain of Sup35 can interfere with the protein’s incorporation into [*PSI*^+^] fibers. Unincorporated Sup35 molecules restore the efficiency of translation termination, making [*PSI*^+^] colonies redder. The extent of interference depends on the interaction between the mutant and the fiber type. For a given fiber structure, different mutants are sequestered differently (or not at all), causing distinct changes in the redness of the yeast colony. The changes form a pattern, which is characteristic of the specific structure. 

The implementation of variant typing was described previously [18,26]. Briefly, 10 plasmids were prepared, all containing a *hphMX* marker [32]. The first five plasmids expressed wild-type Sup35 or single mutants (Q15R, S17R, G44R, or G58D) in addition to endogenous, chromosome-expressed Sup35 to cause variant-specific changes in colony color. The second half of the plasmids expressed fusion proteins consisting of Sup35 N-terminal fragments (Sup35(1-40) or Sup35(1-61)) or mutants (Sup35(1-61)(G20D), (Q23P), or (Q23P & N27P)) in front of the green fluorescent protein (GFP). The fusion proteins variant-specifically labeled [*PSI*^+^] particles. The 10 plasmids were each transformed into prion-free cells whose *HSP104* allele was the same as the colony to be typed and the mating type the opposite. Variant typing was performed by cell mating. Diploids were selected on YPD plates containing G418 and hygromycin. GFP labeling was observed by fluorescence microscopy as described [26].

## 3. Results

### 3.1. Propagation with Hsp104^T160M^

The 23 variants used in this investigation were VH, VK, VL, W8, UnS, Kbar, S17R*, A1, A2, A3, A4, A5 (A1-A5), B1-B6, C1, C2, D1, D2, and D3 [18]. Yeast cytoplasm containing a variant was introduced by cytoduction into prion-free recipients expressing Hsp104^T160M^. Control experiments with recipients expressing wild-type Hsp104 were performed in parallel. The resulting [*PSI*^+^] cytoductants were then variant-typed by a refined procedure (see Materials and Methods). 

We have performed the experiment with VH, VK, and VL previously. VK and VL quickly vanished in cells expressing Hsp104^T160M^, and VH could propagate there but was unstable and easily lost in mitosis. No different variant ever emerged [26]. 

Before discussing the results for other variants, I will address a technical issue in the experimental setup. There are several very weak variants, A2, A3, B1, B3, B4, B6, and D3, that do not propagate well in wild-type cells (Table 1) [18]. However, they are stably maintained in engineered cells whose *SUP35* gene is moved from the chromosome to a centromere-based low-copy-number plasmid [18]. The modification slightly increases the prion protein expression, favoring the growth of [*PSI*^+^] fibers. The modified cell was used as the cytoduction donor to transmit most of the variants. The recipient cells were unmodified. 

The results of the cytoduction experiment are shown in Table 1. Seven variants did not propagate in Hsp104^T160M^-expressing cells (hereafter referred to as T160M cells). For the remaining variants that did, I determined if the variant type remained the same in serial propagation. Two cytoductants from each variant were streaked on agar plates to isolate 10 single colonies for variant typing (five for each cytoductant). One from each lineage was streaked, and sub-colonies were typed again. The procedure was repeated. The experiment revealed unsteady variants that were stabilized with Hsp104^T160M^ and uncovered two mis-templating variants, hitherto incorrectly regarded as faithful-propagating (Table 2). They are discussed below.

#### 3.1.1. Variants Stabilized with Hsp104^T160M^

(a) Stabilization against [PSI+] Curing

B3, B4, and B6 are among the weak [*PSI*^+^] variants that require episomal Sup35 expression to efficiently propagate. They, however, propagated well with Hsp104^T160M^ in cells with normal Sup35 expression (Table 1 and Table 2). The result suggested that the prion-curing activity of wild-type Hsp104 destabilized them in native cells unless more Sup35 was expressed to enhance fiber growth (Hsp104^T160M^ and Hsp104^WT^ are expressed at similar cellular abundance; see ref. [26]).

(b) Stabilization against Transmutation

**Figure 1:**
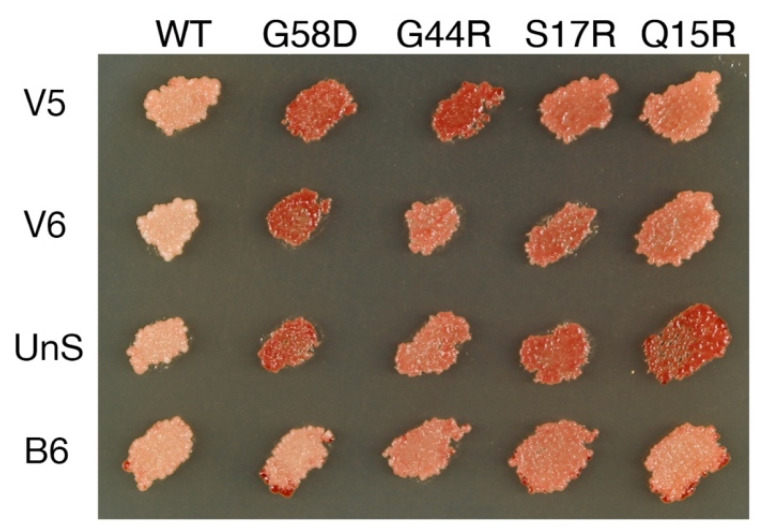
Transmutable [*PSI*^+^] variants stabilized in cells expressing Hsp104^T160M^. Variants are propagated in cells expressing Hsp104^T160M^ and wild-type Sup35 from the chromosome. Different colony color patterns are observed when Sup35 single mutants (labeled on the top) are co-expressed from a plasmid. For V5, the color change is, from left to right, (−, ++, ++, +, +). V6: (−, ++, +, +, +); UnS: (−, ++, +, ++, ++); B6: (−, −, +, +, +). See Materials and Methods for an explanation of the variant typing method.

B6 (sustained with higher Sup35 expression, see the preceding paragraph) and UnS are very mutable when propagated with wild-type Hsp104 [18] but appear faithful-propagating in T160M cells (Table 2, yellow; Figure 1). The variants mis-template VK or VH structures, which cannot efficiently propagate with Hsp104^T160M^, thus the stabilization.

#### 3.1.2. Mis-Templating Variants Revealing Their True Nature 

**Figure 2:**
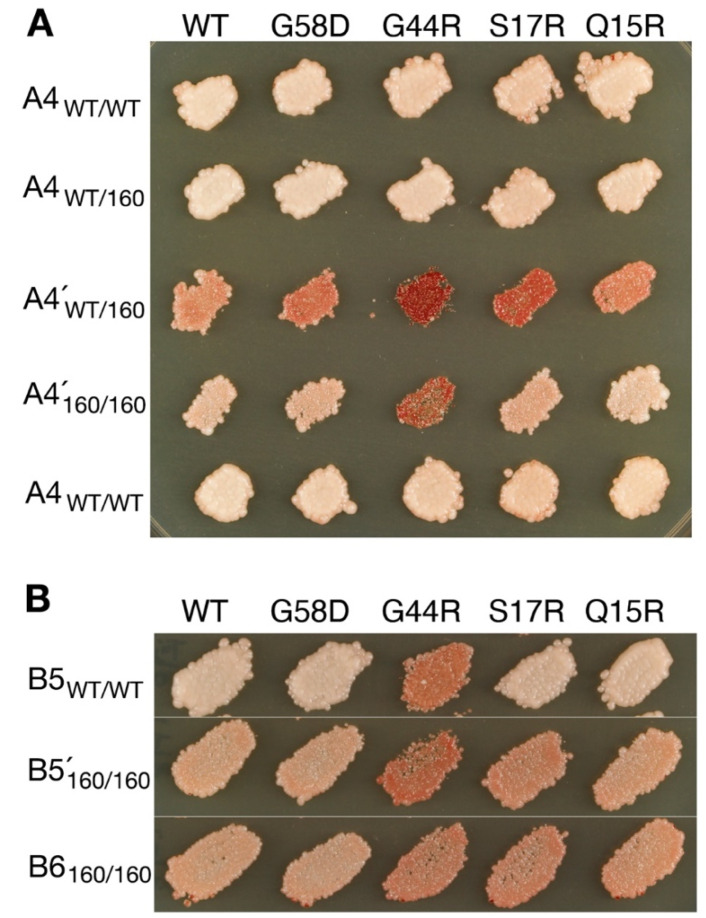
Transmutable [*PSI*^+^] variants revealed in cells expressing Hsp104^T160M^. (**A**) The A4 variant becomes A4′ in cells expressing Hsp104^T160M^ as indicated by distinct patterns of colony color in variant typing. In support of their separate identities, A4 and A4′ are shown to remain distinguished in the same Hsp104^WT/T160M^ background (WT/160). Variant typing is as described in Figure 1. WT/WT and 160/160 indicate Hsp104^WT/WT^ and Hsp104^T160M/T160M^ homozygotes, respectively. (**B**) B5 becomes B5′. The latter is likely a mixture of B5 and B6, as revealed by back-transfer to a WT background (see Table 3).

A4 and B5 seemed to propagate faithfully in wild-type cells but transmuted during serial propagation with Hsp104T^160M^ as judged by different response patterns in variant typing (Table 2, red; Figure 2). The new types, denoted A4′ and B5′, respectively, were introduced back to cells that expressed wild-type Hsp104 from the chromosome and slightly overexpressed Sup35 from a plasmid. A4´ gave rise to mostly A4 colonies and a barely visible VH sector in an otherwise [*psi*^−^] colony, and B5′ gave both B5 and B6 colonies (Table 3).

A4 and A4′ were reconfirmed to be discrete. They were each introduced into Hsp104^WT/160M^ heterozygotes and variant typed. The response patterns remained distinct in the identical background, thus excluding the possibility that they were merely two manifestations of the same element (i.e., one in wild-type cells, and the other in T160M; Figure 2). The two variants might be interconvertible but dominated in separate environments. 

B5′ was likely a mixture of B5 and B6. The transition from B5 to B5′ could be understood in terms of relative fitness between the pair. B5 was less stable in T160M cells, as indicated by significant curing during serial propagation (not shown). In contrast, as described above, B6 did not propagate well with wild-type Hsp104 but was stabilized with Hsp104^T^^160M^. The opposite changes in strength allowed B6 to break out from the dominance of B5. That B5 mis-templated B6 seems to implicate a closer relationship between the two, which is consistent with the fact that they are originally derived from the same ancestor [18]. 

#### 3.1.3. Variants That Did Not Change

With the exception of A1, all remaining variants were significantly destabilized in T160M cells. When cells harboring the variants were streaked on agar plates, most of the colonies arising were red (i.e., lost [*PSI*^+^]). (However, it was still possible to pick out [*PSI*^+^] colonies/sectors to streak on agar plates again, sometimes with the help of a dissection microscope). No mis-templating was observed except C2, which gave rise to several colonies propagating VH, also becoming destabilized, giving many red colonies when streaked on agar (Table 2).

### 3.2. Propagation with Excess Hsp104

I next tested if there were mis-templated structures that required excess Hsp104 to fragment, similar to a special case reported by Borchsenius et al. [33]. Experiments were first performed with W8, VH, VK, and VL. Significant Hsp104 overexpression was needed to weaken W8 and VH. For this, a strong copper-inducible *CUP1* promoter was employed. For VK and VL, Hsp104 was overexpressed with the native promoter from a high-copy-number plasmid. The treatments resulted in [*PSI*^+^] curing for about half of the population after overnight growth in liquid media. Cultures were streaked on agar plates to isolate single [*PSI*^+^] colonies for variant typing while Hsp104 overexpression continued. Two single non-red colonies each were streaked, 10 sub-colonies were typed again (five from each of the two), and the procedure was repeated several times. No new variants were observed (Table 4). 

Other variants were transferred by cytoduction to wild-type cells that overexpressed Hsp104 from a multi-copy plasmid. Only 11 variants were able to produce [*PSI*^+^] cytoductants (see Table 1). Among them, all of the UnS cytoductants were of the VH type; B4 and B6 gave VK only, and C2 VH. They were not analyzed further. Cytoductants from the remaining variants, not mutated yet, were streaked and typed successively (Table 4). Most variants appeared significantly weakened by excess Hsp104 (when cells were streaked on agar, most of the colonies arising lost [*PSI*^+^]), but only D1, D2, and hardly A5 showed mis-templating:

(a) D1 and D2 occasionally mis-templated VK and VH, respectively. The mutability of the former was already observed in mitotic cells without Hsp104 overexpression [18]. 

(b) Out of 49 colonies receiving A5 cytoplasm, there was a single one propagating VH and the rest A5. 

### 3.3. More Variants Were Induced De Novo in T160M Cells

After experimenting with the 23 variants isolated from wild-type cells, I investigated whether there were still more variants that only stably propagated with Hsp104^T160M^, and if so, how they mutated in the wild-type cell. Sup35 overexpression in T160M cells was previously used to induce V2–V4 [26]. To increase the efficiency of discovery, Sup35(S17R) was used here to induce new [*PSI*^+^] variants as V2-V4 propagated less well with Sup35(S17R), thus unlikely to be excessively generated. The S17R mutant has been successfully used to induce the S17R* variant in the wild-type background previously, and it is capable of forming two novel amyloid structures in vitro [18,34]. The plasmid carrying Sup35(S17R) was removed after transient overexpression. Candidate [*PSI*^+^] colonies, now solely supported by the native Sup35 protein, were purified by repeated streaking. Two new variant types, named V5 and V6, were identified (Figure 1). They faithfully propagated in T160M cells (Table 2, yellow), but disappeared when cytoduced to wild-type cells. The majority of the V5 cytoductants propagated VK instead. In contrast, most of the V6 cytoductants lost [*PSI*^+^]; only a few VK colonies showed up (Table 1).

## 4. Discussion

Different balances of cellular strengths in severing and trimming prion fibers can lead to the selection of distinct sets of [*PSI*^+^] variants [26,35]. The current investigation uncovered two mis-templating variants, namely A4 and B5, which appeared to propagate faithfully in wild-type cells because the structures they mis-templated were suppressed (Table 1 and Table 2, red variants). There were also transmutable variants propagating faithfully in T160M cells but revealing mis-templating propensities in wild-type cells, such as UnS and B6 as well as V5 and V6 (yellow variants in Table 1 and Table 2; Figure 1).

Based on mutability, the 23 variants can be classified into three groups: (I) Faithful-propagating: VH, VK, VL, W8, and three other variants (B2, C1, and S17R*) have never been observed to spontaneously mutate (colored blue in Table 1, Table 2 and Table 4; ref. [18]). (II) Marginally transmutable: Three variants (A5, B3, and D3) hardly mutated. For each, in all experiments combined, only a single colony was observed to propagate a different variant (VH, VK, and VH, respectively) (Table 1, Table 2, Table 4 and Table S1, and Table 3 of ref. [18]). (III) Transmutable: the remaining 13 variants were clearly transmutable. However, with the exception of A4 and B5, they mostly changed into VH, VK, or VL, which do not form “prion clouds” [19]; the range of mis-templated structures appeared quite limited (Table 1, Table 2, Table 3, Table 4 and Table S1; ref. [18]). The classification reaffirms our view that the fidelity of prion replication is variant-dependent; seeding is not always full of errors.

There are similarities and differences between [*PSI*^+^] variants and PrP^SC^ strains. For example, sustained propagation of the mouse-adapted 22L strain in cell culture can cause the emergence of a new prion conformation, which loses the ability to replicate in hosts treated with swainsonine, an α-mannosidase II inhibitor. However, the new strain can sometimes mutate back spontaneously to regain the drug resistance [8,36]. Similarly, interconvertible variants were observed in yeast, such as A4 and A4′ reported here; their cellular fitness was influenced by the activity of the heat shock protein Hsp104. Swainsonine interrupts protein glycosylation and induces stress responses in the cell, which may likewise result in selective suppression of strains. One of the major differences between the two prion systems is post-translational modifications of the mammalian prion protein, which generate chemical diversity in cells [37]. Seeding may no longer be considered as homotypic if the modification poses different structural or kinetic barriers to force conformational changes. Binding of small molecules to the mammalian prion could also modify the substrate or the template to cause replication errors, as exemplified by the report that in vitro PrP^SC^ seeding in the presence of phosphatidylethanolamine causes three different strains to change into a common one [38]. Nevertheless, when considering homotypic propagation, the mutability of mammalian prion strains as a whole might not be too different from their yeast counterparts, which, as shown here, do not profusely form “clouds” of different structures. The recent determination of the atomic structures of rodent prions clearly indicates that conformational uniformity of prion samples is achievable [39,40,41].

Our results may rationalize why VH, VK, and VL are by far the most frequently isolated [*PSI*^+^] variants. The lack of structural transmutation suggests that each of them is situated in a local free energy minimum with high transitional barriers. The observation that most transmutable variants ended up generating them indicates that there are many kinetic pathways that lead to the energy minima. The effects combined could make them prevalent. Future studies comparing the folding of VH, VK, VL, and the transmutable variants will be very important. The information obtained will offer insight into the free energy landscape of amyloids and further reveal how transmutable variants mis-template to converge into just a few stable structures.

## Figures and Tables

**Table 1:** Cytoduction of [*PSI*^+^] variants. Recipient cells are indicated on top.

	Hsp104^T160M^					Hsp104^WT^					Hsp104 Overexpression				
	Same	[*psi*^−^]	VH	VK	VL	Same	[*psi*^−^]	VH	VK	VL	Same	[*psi*^−^]	VH	VK	VL
W8	40	18	0	0	0	54	0	0	0	0	—	—	—	—	—
UnS	30	0	27	0	0	0	40	15	1	1	0	34	16	0	0
Kbar	0	56	0	0	0	0	0	0	2	20	0	49	0	0	0
S17R*	58	1	0	0	0	60	0	0	0	0	4(±)	31	0	0	0
A1	50	4	0	0	0	55	3	0	0	0	0	88	0	0	0
A2	0	53	0	0	0	0	57	0	2	0	0	46	0	0	0
A3	0	59	1	0	0	0	60	0	0	0	0	49	0	0	0
A4	54	0	0	0	0	54	0	0	0	0	3	39	0	0	0
A5	51	3	0	0	0	54	0	0	0	0	34	5	0	0	0
B1	0	53	0	0	0	0	49	0	2	4	0	48	0	0	0
B2	0	53	0	0	0	50	3	0	0	0	18(±)	37	0	0	0
B3	58	0	0	0	0	0	53	0	1	0	0	46	0	0	0
B4	55	0	0	0	0	1	52	0	2	0	0	40	0	12(±)	0
B5	47	8	0	0	0	53	0	0	0	0	39	7	0	0	0
B6	32	22	0	0	0	0	27	1	31	1	0	31	0	1	0
C1	4	20	0	0	0	45	12	0	0	0	0	67	0	0	0
C2	48	0	1	0	0	54	2	0	0	0	0	32	1	0	0
D1	0	56	0	0	0	55	0	0	2	0	1	49	0	1	0
D2	56	2	0	0	0	57	0	0	0	0	44	0	0	0	0
D3	0	60	0	0	0	0	58	0	0	0	0	44	0	0	0
V5	45	2	0	0	0	0	3	2	51	0	—	—	—	—	—
V6	55	0	0	0	0	0	53	0	6	0	—	—	—	—	—

Same: the same type as the donor. VH, VK, and VL: cytoductants propagating VH, VK, and VL, respectively, which were the only mis-templated variant types observed at this stage. (±): colonies containing a significant proportion of [*psi*^−^] cells. –: not done. Hsp104 was expressed from the native promoter; overexpression was achieved by transformation with a multi-copy plasmid carrying the Hsp104 gene. Blue: faithful propagating variants. Yellow: transmutable but stabilized in T160M cells. Red: transmutable but stabilized in wild-type cells. The classification uses additional data from Tables 2–4 as well as Table 3 of reference [18]. A2, A3, B1, and D3 donors, giving few [*PSI*^+^] cytoductants here, were verified to efficiently generate expected [*PSI*^+^] cytoductants in Hsp104^WT^ recipients with higher Sup35 expression (Table S1).

**Table 2:** Serial propagation with Hsp104^T160M^.

	1st Streak	2nd	3rd	4th	5th	6th
W8	10W8	10	10	10	10	10
UnS	10UnS	9	10	10	10	10
S17R*	10S17R*	9	10	10	10	8
A1	10A1	10	9	10	10	9
A4	5A4 + 5A4′	5A4 + 5A4′	10A4′	10A4′	10A4′	10A4′
A5	10A5	10	8	10	8	9
B3	10B3	10	10	10	10	10
B4	10B4	10	10	10	10	10
B5	5B5′	5B5′	5B5′	5B5′	5B5′	5B5′
B6	10B6	10	10	9	10	10
C1	10C1	10	10	10	10	10
C2	8C2 + 2VH	9C2 + 1VH	7C2 + 3VH	8C2 + 2VH	8C2 + 1VH	9C2
D2	10D2	10	5	5	5	5
V5	10V5	10	10	10	10	10
V6	10V6	10	10	10	10	10

Ten non-red colonies from two lineages (five each) are selected and variant-typed for each entry. The number of [*psi*^−^] colonies is (10-X), X representing the number listed in each entry. In most cases, they are white Ade^+^ revertants that lack particulate GFP labeling. The color code is the same as in Table 1. Blue: faithful propagating variants. Yellow: transmutable but stabilized in T160M cells. Red: transmutable but stabilized in wild-type cells. A4′ and B5′ are distinct from A4 and B5, respectively. For A4, a cytoductant produced sub-colonies of the A4′ type at the “first streak”, and the other not so until the 3rd. For B5, one produced B5′ and the other accidentally lost the prion. One of the D2 lineages lost [*PSI*^+^] on the “3rd streak”.

**Table 3:** Reverse cytoduction (Hsp104^T160M^ → ^WT^).

	Colony #1	Colony #2
A4′	15A4, 14[*psi*^-^]	4A4, 1VH, 13[*psi*^-^]
B5′	5B5, 6B6, 12[*psi*^-^]	1B5, 3B6, 6[*psi*^-^]

**Table 4:** Serial propagation with excess Hsp104.

	1st Streak	2nd	3rd	4th	5th
VH	10VH	10	10	10	10
VK	10VK	10	10	10	10
VL	10VL	10	10	10	10
W8	10W8	10	10	10	10
S17R*	10S17R*	9	10	10	10
A4	10A4	10	10	10	10
A5	10A5	10	9A5 + 1VH	9	10
B2	10B2	10	9	5	9
B5	10B5	10	10	9	9
D1	10D1	9D1 + 1VK	9D1 + 1VK	9D1 + 1VK	7D1 + 2VK
D2	6D2 + 3VH	8D2 + 2VH	10D2	9D2 + 1VH	9D2 + 1VH

Ten non-red colonies/sectors from two lineages (five each) are variant-typed. The number of [*psi*^−^] colonies (=10-X) is not listed. Color code is the same as in Table 1. Blue: faithful propagating variants. Red: transmutable but stabilized in wild-type cells.

## Data Availability

Not Applicable.

## Supplementary Materials

The following supporting information can be downloaded at: https://www.mdpi.com/article/10.3390/v14112337/s1, Table S1: Cytoduction of [*PSI*^+^] variants that require higher Sup35 expression for propagation.
