# Supplementary material for: The Mutability of Yeast Prions
Source: Viruses. 2022 Oct 25;14(11):2337. doi: 10.3390/v14112337 (PMC9696419; doi:10.3390/v14112337)
Supplement: Supplementary file 1 [file viruses-14-02337-s001.zip › viruses-1869843-supplementary.pdf]

Table S1. Cytoduction of [*PSI*+] variants (left) that require higher Sup35 expression for propagation.

	Hsp104^{WT} & Sup35 overexpression				
	Same	[*psi*-]	VH	VK	VL
A2	15	4	0	1	0
A3	18	0	2	0	0
B1	13	3	0	4	0
D3	19	1	0	0	0
